# Association of inflammatory biomarkers with new functional morbidity at hospital discharge in children who survive severe sepsis

**DOI:** 10.3389/fped.2025.1519246

**Published:** 2025-03-07

**Authors:** Mallory A. Perry-Eaddy, Walter Faig, Martha A. Q. Curley, Scott L. Weiss

**Affiliations:** ^1^ School of Nursing, University of Connecticut, Storrs, CT, United States; ^2^ Deptartment of Pediatrics, School of Medicine, University of Connecticut, Farmington, CT, United States; ^3^ Pediatric Intensive Care Unit, Connecticut Children’s Medical Center, Hartford, CT, United States; ^4^ Department of Biostatistics, Children’s Hospital of Philadelphia, Philadelphia, PA, United States; ^5^ Department of Family and Community Health, School of Nursing, University of Pennsylvania, Philadelphia, PA, United States; ^6^ Anesthesia and Critical Care Medicine, Perelman School of Medicine, University of Pennsylvania, Philadelphia, PA, United States; ^7^ Research Institute, Children’s Hospital of Philadelphia, Philadelphia, PA, United States; ^8^ Department of Critical Care Medicine, Nemours Children’s Hospital, Wilmington, DE, United States; ^9^ Department of Pediatrics, Sidney Kimmel Medical College, Thomas Jefferson University, Philadelphia, PA, United States

**Keywords:** sepsis, inflammation, biomarkers, functional status, pediatric intensive care unit

## Abstract

**Objective:**

New functional morbidity is common in critically ill children who survive sepsis; yet, the underlying biological mechanisms, particularly the impact of inflammation, remain unknown. We sought to test the hypothesis that increased levels of inflammatory biomarkers during the acute phase of pediatric sepsis are associated with new functional morbidity at hospital discharge.

**Methods:**

We conducted a *post hoc* secondary analysis of the *MitoPSe* clinical study, including *N* = 119 critically ill children who survived sepsis. Data collected included demographic and clinical variables and 31 inflammatory biomarkers collected at three distinct timepoints (within days 1–2 of PICU admission, days 3–5, and days 8–14). The primary outcome was new functional morbidity, defined as at least a one-point increase in the pediatric overall performance category from baseline to hospital discharge.

**Results:**

New functional morbidity occurred in 38 children (32%) and was associated with increased plasma levels of interleukin (IL)-6, IL-18, sIL-2Ra, MCP1, IL-8 (CXCL8), sIL-1RII, IL-10, MIP1a, and IL-2r and decreased RANTES (CCL5) (*p* < .001) at all three timepoints. However, after adjusting for differences in chronic comorbid conditions, hospital length of stay, number of organ dysfunctions, and severity of illness, absolute biomarker levels, and trajectories were not significantly different between patients with or without new functional morbidity at hospital discharge.

**Conclusions:**

In this sample of critically ill children treated for sepsis, increased inflammatory biomarker levels and the trajectory of change during the acute phase of pediatric sepsis were not independently associated with new functional morbidity at hospital discharge. Inflammatory biomarker levels likely reflect illness severity and other clinical variables associated with illness. However, these biomarkers may still be useful in identifying patients at risk of developing functional morbidity, despite the lack of causation within this study.

## 1 Introduction

Sepsis is a leading public health problem in children, affecting more than 75,000 children in the United States annually (1, 2). With earlier recognition, infectious source control, and resuscitation, more than 80% of children hospitalized for sepsis now survive (3). However, nearly a third of sepsis survivors who require intensive care suffer new morbidities and struggle to return to their pre-sepsis functional baseline (4–10). In the Life After Pediatric Sepsis (*LAPSE*) study, 35% of pediatric sepsis survivors did not regain their baseline health-related quality of life 1 year after their critical illness, with deficits in physical function being the most common (5).

Despite improved epidemiologic studies characterizing post-sepsis outcomes, there are limited data on the biological mechanisms underlying or risk factors associated with functional morbidities in children who survive sepsis. In adult sepsis survivors, the severity of the inflammatory response during critical illness has been proposed as a mechanism driving new functional morbidity after discharge (11, 12). However, the impact of systemic inflammation on post-sepsis pediatric outcomes beyond mortality has not been well described. To address this knowledge gap in pediatric sepsis, we sought to test the hypothesis that elevated levels of cytokines and chemokines during critical illness are associated with new functional morbidity at hospital discharge.

## 2 Methods

We conducted a secondary analysis of the Mitochondrial Dysfunction in Pediatric Sepsis (*MitoPSe*) study (13, 14). Briefly, *MitoPSe* investigated mitochondrial dysfunction as it pertains to the immune response in children with severe sepsis/septic shock in the pediatric intensive care unit (PICU) between May 2014 and June 2018 (13). *MitoPSe* included the measurement of inflammatory cytokines/chemokines at three timepoints during the acute phase of illness in 166 critically ill children aged <18 years with severe sepsis and/or septic shock, as defined by the International Pediatric Sepsis Consensus Conference (IPSCC) criteria (15). In this *post hoc* secondary analysis, we include the subset of *MitoPSe* participants who survived hospital discharge and provided consent to participate in future research (Supplementary Figure S1). This secondary analysis was determined to be exempt from human subject research by the Children's Hospital of Philadelphia Institutional Review Board.

### 2.1 Clinical data

Clinical data were obtained using manual chart review from the electronic health record (EHR) and the Virtual PICU Systems (VPS), LLC (16). Variables included demographics, chronic comorbid conditions, and acute phase lab and clinical data. The severity of illness was assessed on admission as cumulative severity of organ dysfunction defined by the pediatric logistic organ dysfunction (PELOD) (17) score and overall illness severity scores, including Pediatric Risk of Mortality (PRISM-III) (18) and Pediatric Index of Mortality (PIM2) abstracted from VPS (19). Organ dysfunction and organ dysfunction days were also defined in accordance with the IPSCC (15)*.* Measures of healthcare resource utilization included transfer to an inpatient rehabilitation facility, need for new equipment and medical services at hospital discharge (i.e., homecare nursing, outpatient needs), change in functional status scale (FSS) between baseline and hospital discharge, and hospital readmission within 6 months.

The primary endpoint was new functional morbidity at hospital discharge, defined as at least a one-point increase in the pediatric overall performance category (POPC) score from pre-illness baseline to hospital discharge. POPC is a six-point Likert scale, ranging from 1 (normal) to 6 (death) (20). Pre-illness POPC was obtained from VPS, and hospital discharge POPC was calculated using data available within the EHR clinical notes and discharge summaries. Functional status was also assessed by the functional status scale (FSS). The FSS is a Likert scale instrument that assesses functional status in children across six domains: mental status, sensory, communication, motor, feeding, and respiratory function. Each domain is scored from normal to severely impaired, providing a total score that reflects the child's overall level of functional disability. The total scores range from 6 (normal) to 30 (severely impaired) (21). The secondary outcomes included resource utilization assessed by discharge disposition (e.g., transfer to inpatient rehabilitation facility), need for readmission at 6 months, or new equipment and medical services (i.e., homecare nursing, outpatient needs) required at hospital discharge.

### 2.2 Inflammatory biomarkers

Thirty-one inflammatory biomarkers were assayed as part of the parent *MitoPSe* study and included in this analysis (Supplementary Table S1). Plasma samples were obtained at three pre-specified time points:

1. Days 1–2 of PICU admission,
2. Day 3–5 (and at least 2 days after the first sample)
3. Between days 8 and 14.

While the initial plasma sample was obtained in the PICU, subsequent samples could be obtained in the PICU or other inpatient locations. Inflammatory biomarkers were assayed in duplicate using commercially available kits, as previously described and outlined in Supplementary Table S1 (13).

### 2.3 Statistical analysis

Analyses were conducted in SAS (Version 9.4; SAS Institute Inc., Cary, NC, USA). Continuous variables are presented using mean (±SD) or median (IQR) and categorical variables as frequencies and percentages. Patient characteristics and clinical measures were compared using a two-sample *t*-test or the Wilcoxon rank sum test for continuous variables and the chi-square test for categorical variables. Extreme outliers were assessed and removed. While all enrolled children had blood specimens collected during at least one time point, we anticipated non-random missingness of biomarkers (e.g., later time points absent due to rapid clinical recovery and short hospital length of stay). To account for the non-random missingness of data, we used mixed-effects logistic regression models. Each biomarker was log-transformed to normalize its distribution. The association of log-scale biomarker levels with the primary outcome was first assessed by mixed-effects regression using the Holm–Bonferroni correction for multiple comparisons. Biomarkers associated with new functional morbidity were plotted over time, dichotomized by the presence of new functional morbidity. For the selected biomarkers, mixed-effects regression was then performed with the inclusion of timepoint and POPC change interaction term to determine if the trajectory of biomarkers differed between children with and without new functional morbidity. Demographic and clinical covariates were considered potential confounders if the association with the primary outcome was significant (*p* < 0.05). The severity of illness was forced into the model, using either PELOD or PRISM. For PELOD, trichotomous variables of low (<10), moderate (<20), and high (≥20) severity of organ dysfunction/illness severity were utilized (17), whereas PRISM was modeled as a continuous variable indicative of mortality risk.

## 3 Results

A total of 119 sepsis survivors were included in this analysis. Patient characteristics for those with and without new functional morbidity at hospital discharge are shown in Table 1. Of the 119 survivors, 38 (32%) experienced new functional morbidity at hospital discharge. There were no differences in age, sex, race, severity of illness, lactate, primary pathogen, or site of infection between groups, but patients who developed new functional morbidity were more likely to have chronic comorbid conditions (29% vs. 11%, *p* = 0.02), exhibited a higher mean number of organ dysfunctions on day 1 [3 (SD ± 1.2) vs. 2.2 (SD ± 1.1); *p* < 0.01], exhibited a higher percentage of organ dysfunction on day 14 (58% vs. 20%, *p* < 0.01), and were more likely to have hospital-acquired infections at 28 days (24% vs. 9%, *p* = 0.02) than those without new functional morbidity. Patients with new functional morbidity also had significantly longer median PICU lengths of stay [13 (IQR 9, 30) days] than those without new morbidity [8 (3, 14) days; *p* < 0.001].

**Table 1:** Patient characteristics.

Characteristic	New functional morbidity present	New functional morbidity absent	*P*-value
	*N* = 38	*N* = 81	
Age—years, mean (SD)	8.6 (5.8)	9.1 (5.5)	0.63
Gender—female, *N* (%)	13 (34.2)	42 (51.9)	0.07
Race, *N* (%)			0.69
White	19 (50)	43 (51.9)	
Black	8 (21.1)	21 (25.9)	
Other	11 (29)	18 (22.2)	
PICU length of stay, median (IQR)	13 (9, 30)	8 (3, 14)	<0.01
Hospital length of stay, median (IQR)	28 (17, 61)	13 (8, 24)	<0.01
Pre-PICU health status, *N* (%)			
Previous comorbid conditions	11 (28.9)	9 (11.1)	0.02
Oncological condition	8 (21.1)	10 (12.4)	0.22
Severity of illness on admission			
PRISM-III, mean (SD)	12.8 (9.6)	11.5 (7.8)	0.43
PIM2, mean (SD)	6.5 (11.7)	4.7 (9.7)	0.38
PELOD, *N* (%)			0.79
Low (0–9)	3 (7.9)	4 (4.9)	
Moderate (10–19)	37 (71.1)	61 (75.3)	
High (20+)	8 (21.1)	16 (19.8)	
Sepsis clinical course			
Lactate, highest value, mean (SD)	4.5 (3.8)	3.3 (2.2)	0.09
Primary pathogen, *N* (%)			0.58
Bacterial	17 (46)	30 (37)	
Viral	10 (27)	22 (27.2)	
No pathogen identified	10 (27)	29 (35.8)	
Primary site of infection, *N* (%)			0.40
Blood (bacteremia)	9 (23.7)	12 (14.8)	
Respiratory (pneumonia, bronchiolitis)	11 (29)	34 (42)	
Other	10 (26.3)	23 (28.4)	
Unknown	8 (21.1)	12 (14.8)	
Healthcare-acquired infection, 28 days, *N* (%)	9 (23.7)	7 (8.6)	0.02
Number organ dysfunctions, day 1, median (IQR)	3.0 (2–4)	2 (2–3)	<0.01
Organ dysfunction, day 14, *N* (%)	22 (57.9)	16 (19.8)	<0.01
Baseline functional status, median (IQR)			
Pediatric overall performance category (POPC)	2 (1, 2)	2 (1, 4)	<0.01
Functional status scale (FSS)	6 (6, 9)	10 (6, 15)	0.01

Variables: PICU, pediatric intensive care unit; PRISM-III, pediatric risk of mortality III; PIM2, pediatric index of mortality 2; PELOD, pediatric logistic organ dysfunction, POPC, pediatric overall performance category, FSS, functional status scale.

Most children had mild functional disability at baseline with a median baseline POPC of 2 (1, 3), but there was less variability in baseline POPC in children who developed new functional morbidity [2 (1, 2)] compared to those without [2 (1, 4)], *p* < 0.01; Table 2). In children with new morbidity, the magnitude of change in POPC was most often identified as an increase of 1 point (71%) from baseline to discharge. The median magnitude of change in POPC is detailed in Table 2. Patients with new functional morbidity were also more likely to experience an increase in FSS ≥ 3 from baseline to hospital discharge. Patients with new functional morbidity more often required new equipment (e.g., feeding tube), new medical services, and new outpatient services (Table 2; Supplementary Table S2) than patients without new functional morbidity. The proportion of readmissions at 6 months was not different between groups, but planned readmissions were more common among patients with new functional morbidity.

**Table 2:** Functional assessment at hospital discharge.

Measure, *N* (%)	New functional morbidity present	New functional morbidity absent	*P*-value
	*N* = 38	*N* = 81	
Change in POPC from baseline			
0	0	81 (100)	
1	27 (71)	–	
≥2	11 (29)	–	
Change in FSS from baseline			<0.01
<3	17 (45)	79 (97)	
≥3	21 (55)	2 (3)	
Readmission at 6 months	21 (55)	47 (58)	0.78
Cause of readmission^a^			
Infection	8 (38)	33 (70)	<0.01
Non-infectious acute	13 (62)	42 (89)	<0.01
Non-infectious planned	10 (48)	1 (2)	<0.01
New medication(s)	33 (87)	60 (74)	0.12
New medical equipment	30 (79)	21 (26)	<0.01
New subspecialty service	35 (92)	55 (68)	<0.01
New ancillary service	34 (90)	21 (26)	<0.01

Variables: POPC, pediatric overall performance category; FSS, functional status scale.

^a^ Fischer's exact test.

**Figure 1:**
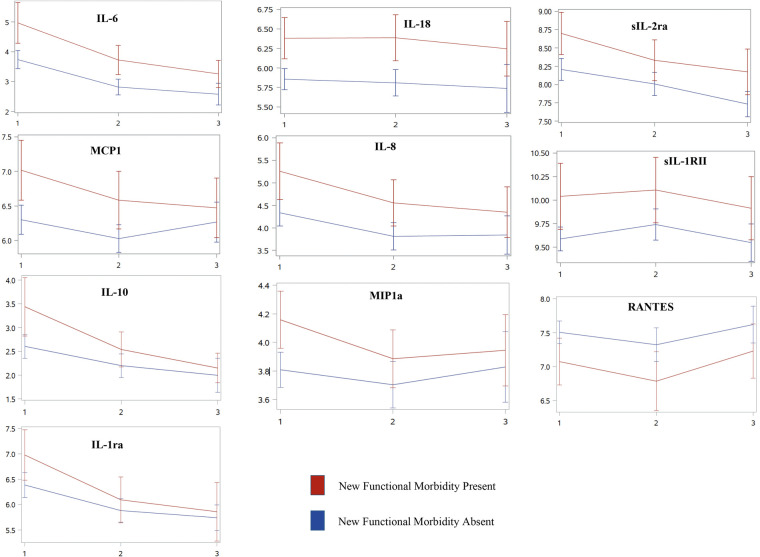
Inflammatory biomarker trajectories associated with new functional morbidity at PICU discharge over time. Unadjusted trajectories of log-transformed mean inflammatory biomarker levels [IL-6, IL-18, sIL-2Ra, MCP1, IL-8 (CXCL8), sIL-1RII, IL-10, MIP1a, RANTES (CCL5), and IL-2r] that are associated with new functional morbidity across three distinct timepoints, (1) days 1–2 of PICU admission, (2) days 3–5, and (3) between days 8 and 14. With the exception of RANTES, each biomarker followed similar trajectories with elevated biomarker levels in those who develop new morbidity at PICU discharge compared to those who do not. After adjusting for clinical variables, inflammatory biomarkers no longer remained associated with new functional morbidity.

Ten biomarkers were associated with new functional morbidity after correction for multiple comparisons in unadjusted analyses: interleukin (IL)-6, IL-18, sIL-2Ra, MCP1, IL-8 (CXCL8), sIL-1RII, IL-10, MIP1a, RANTES (CCL5), and IL-2r (Table 3). At each time point, log-transformed levels of these biomarkers were increased in those with new morbidity, except for RANTES which exhibited lower levels, compared to those without new morbidity (Figure 1). However, the overall trajectory of these biomarkers was not different in unadjusted analyses between those with and those without new functional morbidity.

**Table 3:** Association of biomarkers with new functional morbidity and trajectory over time.

Biomarker	Unadjusted *P*-values			Adjusted *P*-values^a^		
	New functional morbidity	Change over time	Change over time by group	New functional morbidity	Change over time	Change over time by group
IL-6	<0.001	<0.001	0.35	0.14	<0.001	0.44
IL-18	0.002	0.38	0.81	0.08	0.50	0.74
sIL2-Ra	0.007	<0.001	0.15	0.10	<0.001	0.09
MCP-1	0.02	0.006	0.12	0.24	0.001	0.28
IL-8	0.01	0.001	0.48	0.74	<0.001	0.56
sIL-1 RII	0.02	0.006	0.76	0.81	0.004	0.71
IL-10	0.05	<0.001	0.09	0.68	<0.001	0.12
MIP-1α	0.07	0.002	0.22	0.34^b^	0.001^b^	0.27^b^
RANTES	0.02	0.01	0.81	0.38^b^	0.008^b^	0.83^b^
IL-2R	0.03	< 0.001	0.22	0.06	<0.001	0.27

Inflammatory biomarkers: IL-6, interleukin-6; IL-18, interleukin-18; sIL-2ra, soluble interleukin-2 receptor alpha; MCP-1, monocyte chemoattractant protein-1, IL-8, interleukin-8, sIL-1RII, soluble interleukin-1 receptor II; IL-10, interleukin-10; MIP-1α, macrophage inflammatory protein-1 alpha; regulated upon activation, RANTES, normal *T* cell expressed and secreted, GCSF, granulocyte colony-stimulating factor, IL-2R, interleukin-2 receptor, IL-1RA, interleukin-1 receptor antagonist.

New functional morbidity is defined as ≥1 increase in the pediatric overall performance category from pre-PICU baseline to PICU discharge. Change over time in the model refers to the pre-specified timepoints (within 1–2 days of PICU admission, days 3–5, and days 8–14). Change over time by group includes both the functional morbidity (yes/no) and change over time variables as the interaction term.

^a^ Models adjusted for comorbid conditions, hospital length of stay, pediatric logistic organ dysfunction (PELOD) score category (low/moderate/high), and number of organ dysfunction day 1.

^b^ PRISM score (numeric) replaces PELOD in adjusted models.

Based on differences in characteristics between groups, we included chronic comorbid conditions, hospital length of stay, number of organ dysfunctions on day 1, and either PELOD or PIM2 score as covariates in separate mixed-effects linear regression models to assess for differences in biomarker levels and trajectory between groups using time and group assignment as an interaction term. After adjusting for these covariates, none of the 10 inflammatory biomarker levels were associated with new functional morbidity. Although most biomarkers continued to demonstrate a significant decrease over time, there were no differences in biomarker trajectories over time between groups (Table 3).

## 4 Discussion

In this *post hoc* secondary analysis of 119 critically ill children who survived sepsis, one-third experienced new functional morbidity at the time of hospital discharge. Our data show that children who survive sepsis have increased levels of cytokine and chemokine during their acute phase of illness and these levels are associated with the development of new functional morbidity. However, this association did not hold after controlling for clinical confounding variables. Specifically, patients who developed new functional morbidity presented with a higher burden of organ dysfunction were more likely to experience healthcare-acquired infections and had a longer ICU length of stay. Despite, this early trajectories of biomarker levels may be helpful as a proxy for later clinical variables which are associated with new morbidity.

Our morbidity findings are consistent with those in the *LAPSE* study, where one-third of children continued to experience functional morbidity up to 6- months after hospital discharge (6, 22). Identifying potential risk factors during the acute phase of sepsis may aid in improved prognostication of those children who may be at increased risk of having new functional morbidity after PICU discharge.

Patients who developed new functional morbidity generally had higher levels of circulating cytokines and chemokines than patients who did not develop new morbidity. However, inflammatory biomarkers decreased in all patients, and the downward trajectory did not differ by risk for new functional morbidity. Moreover, after controlling for differences in baseline characteristics and illness severity, inflammatory biomarker levels no longer differed between those with and without new functional morbidity at baseline. While the biomarkers themselves do not have independent predictive value when adjusted for confounding variables, they may act as surrogates for covariates like illness severity potentially contributing to new functional morbidity.

Within survivors of the *MitoPSe* cohort, the development of new functional morbidity at hospital discharge was associated with ten inflammatory biomarkers measured during a child's acute illness - IL-6, IL-18, sIL-2Ra, MCP1, IL-8 (CXCL8), sIL-1RII, IL-10, MIP1a, RANTES (CCL5), and IL-2r. Although children generally experienced improved levels of inflammatory biomarkers over time irrespective of the development of new functional morbidity, most biomarker levels were higher at each time point in those who developed new functional morbidity compared to those who did not.

Only one of the ten associated biomarkers, RANTES, consistently had an inverse relationship between groups regarding log-biomarker levels of inflammation. Lower levels of RANTES were observed in those with new functional mobility at each time point compared to those without. These findings are consistent with previous pediatric research and the severity of the illness in infectious diseases (23, 24). This is also found in adult and non-neonatal pediatric populations, which have concluded that low levels of RANTES (CCL5) have increased the predictive value of mortality (23, 25). Neonatal-specific research concluded that downregulation of RANTES over time may predict the development of sepsis-induced disseminated intravascular coagulation (24). In adults, a low level of RANTES is independently associated with cardiac mortality (26). Though to our knowledge while the association between RANTES (CCL5) and mortality is established, its association with morbidity is unknown. RANTES is important for immune response activation, including being a key chemoattractant of monocytes and lymphocytes during infection. Low levels may leave an individual vulnerable to an impaired immune response and re-infection. As such, low levels and downregulation of RANTES may be an important prognostic target for early morbidity recognition throughout the acute period of illness in critically ill children.

Contradictory to RANTES decreased levels, in adult ICU patients, increased inflammation and new morbidity yield similar results. While early high levels of biomarkers may have clinical significance in determining overall treatment plans, the association between early inflammatory biomarker levels and poor outcomes is inconsistent. A secondary analysis of the ALTOS trial included adult ICU survivors with acute respiratory distress syndrome and sepsis and identified subphenotypes (hypo- vs. hyper-inflamed). Peripheral inflammatory biomarkers were drawn, and assessed, early in the critical illness (at the time of trial randomization) (27). Those with hyperinflammatory subphenotypes experienced a higher overall 12-month mortality rate; yet, physical, cognitive, and mental outcomes at 6 and 12 months were similar between groups. A similar lack of association is highlighted in Brummel et al., exploring the association between elevated inflammatory biomarkers, cognitive impairment, and disability among adults (28). However, literature shows that follow-up of biomarkers beyond the immediate critical illness may aid in understanding morbidity development. In older adults, an elevated level of C-reactive protein, a non-specific inflammatory marker associated with IL-6, at and beyond ICU discharge is associated with functional impairment after an ICU admission (21), thereby indicating the potential need for clinicians to look beyond single timepoint biomarker level associations and fully assess the trajectory of biomarker levels, including those at ICU discharge. Such biomarkers and analyses may be surrogate indicators of post-ICU morbidity.

Overall, our findings support the use of early identification of those who may be at increased risk of new functional morbidity after discharge, irrespective of biomarker levels. Early identification and intervention may aid in recovery. In multivariate analyses, while biomarker levels were not significant between groups, several pre-PICU and acute phase factors were associated with increased risk of new morbidity—the presence of chronic comorbid conditions, increased PICU and hospital length of stay, and the number of organs with dysfunction on day 1 of sepsis illness. Within our cohort, there is also evidence of increased resource utilization including new equipment, medical services, outpatient services, and readmission within 6 months after PICU discharge in those with new functional morbidity. These risk factors and outcomes have been previously discussed in the PICU literature (29). As such, clinicians can provide continued support and resources for at-risk children and families throughout the critical illness period to augment a child's recovery.

### 4.1 Limitations

A primary limitation of our study is the secondary use of existing data and retrospective data collection. Inherent to a retrospective chart review, there is the possibility of inaccurate, incomplete, or missing data. To address this concern, a single reviewer (MP-E) conducted a chart review of discharge and follow-up data to improve the reliability of the data. Future studies in post-sepsis care should include prospective data collection with follow-up data beyond 6 months in critically ill children who survive sepsis to understand the impact of critical illness on long-term functional morbidity beyond PICU discharge. To minimize any methodological errors while completing a retrospective chart review, standards set forth by Vassar and Holzmann were taken into consideration (30).

The POPC is a seminal, widely used, but crude measure of PICU functional outcomes (20, 21). Within this manuscript, we define our primary outcome of functional morbidity as any worsening in the POPC score, which is consistent with previous PICU studies (31, 32). Despite POPC's lack of granularity, studies have shown that POPC may be useful in assessing the developmental and functional status and supporting its use. In a study by Fiser et al., there was a significant association between POPC scores and the Bayley Psychomotor Developmental Index scores and Vineland Adaptive Behavior Scales scores (*p* < .0001), thus serving as a potential proxy for in-depth developmental and functional testing, which may not always be feasible in critically ill sepsis survivors.

In addition, this secondary analysis of the *MitoPSe* cohort was underpowered to detect smaller effects across those with and without new functional morbidity. As such, definitive conclusions cannot be drawn regarding these specific inflammatory biomarkers. A larger sample size of sepsis survivors is necessary to understand the potential cytokine trajectories in a similar cohort. As such, we were unable to assess absolute cytokine values/time to establish clinically relevant cytokine cut-points that, together, provide the most accurate discrimination of new functional morbidity.

## 5 Conclusion

Children who survive sepsis are at risk for new functional morbidity and increased resource utilization after discharge. Within our cohort, most inflammatory biomarkers remained elevated throughout the acute phase of illness in children who developed new morbidity, except RANTES. Despite this trend, the overall biomarker trajectories were non-significant, as inflammation resolution trajectories followed similar patterns between those who developed new morbidity and those who did not. The inflammatory milieu associated with new functional morbidity was confounded by more easily measurable clinical and illness severity variables, so we did not identify independent values for the use of cytokines/chemokines to predict or identify the risk of new functional morbidity in this study. Future studies with larger cohorts of PICU survivors, focused on the association of new functional morbidity and inflammatory biomarker profiles within the acute phase of illness and the use of more sensitive outcome measures may be useful to better assess for risk of post-PICU outcomes.

## Data Availability

The original contributions presented in the study are included in the article/Supplementary Material, further inquiries can be directed to the corresponding author.

## References

[1] Randolph AG McCulloh RJ. Pediatric sepsis. Virulence. (2014) 5(1):179–89. 10.4161/viru.27045 24225404 PMC3916372

[2] Hartman ME Linde-Zwirble WT Angus DC Watson RS. Trends in the epidemiology of pediatric severe sepsis*. Pediatr Crit Care Med. (2013) 14(7):686–93. 10.1097/PCC.0b013e3182917fad 23897242

[3] Balamuth F Weiss SL Neuman MI Scott H Brady PW Paul R Pediatric severe sepsis in US children’s hospitals. Pediatr Crit Care Med. (2014) 15(9):798–805. 10.1097/PCC.0000000000000225 25162514 PMC4221502

[4] Farris RW Weiss NS Zimmerman JJ. Functional Outcomes in Pediatric Severe Sepsis. Pediatr Crit Care Med. (2013) 14(9):835–42. 10.1097/PCC.0b013e3182a551c8 24108117 PMC4080839

[5] Prout AJ Talisa VB Carcillo JA Angus DC Chang CH Yende S. Epidemiology of readmissions after sepsis hospitalization in children. Hosp Pediatr. (2019) 9(4):249–55. 10.1542/hpeds.2018-0175 30824488 PMC6434975

[6] Zimmerman JJ Banks R Berg RA Zuppa A Newth CJ Wessel D Trajectory of mortality and health-related quality of life morbidity following community-acquired pediatric septic shock. Crit Care Med. (2020) 48(3):329–37. 10.1097/CCM.0000000000004123 32058370 PMC7164680

[7] Prescott HC Angus DC. Enhancing recovery from sepsis. JAMA. (2018) 319(1):62–75. 10.1001/jama.2017.17687 29297082 PMC5839473

[8] Prescott HC Iwashyna TJ Blackwood B Calandra T Chlan LL Choong K Understanding and enhancing sepsis survivorship. Priorities for research and practice. Am J Respir Crit Care Med. (2019) 200(8):972–81. 10.1164/rccm.201812-2383CP 31161771 PMC6794113

[9] Rhodes A Evans LE Alhazzani W Levy MM Antonelli M Ferrer R Surviving sepsis campaign: international guidelines for management of sepsis and septic shock: 2016. Crit Care Med. (2017) 45(3):486–552. 10.1097/CCM.0000000000002255 28098591

[10] Weiss SL Peters MJ Alhazzani W Agus MSD Flori HR Inwald DP Surviving sepsis campaign international guidelines for the management of septic shock and sepsis-associated organ dysfunction in children. Pediatr Crit Care Med. (2020) 21(2):e52–e106. 10.1097/PCC.0000000000002198 32032273

[11] Griffith DM Lewis S Rossi AG Rennie J Salisbury L Merriweather JL Systemic inflammation after critical illness: relationship with physical recovery and exploration of potential mechanisms. Thorax. (2016) 71(9):820–9. 10.1136/thoraxjnl-2015-208114 27118812

[12] Griffith DM Vale ME Campbell C Lewis S Walsh TS. Persistent inflammation and recovery after intensive care: a systematic review. J Crit Care. (2016) 33:192–9. 10.1016/j.jcrc.2016.01.011 26880401

[13] Weiss SL Zhang D Bush J Graham K Starr J Tuluc F Persistent mitochondrial dysfunction linked to prolonged organ dysfunction in pediatric sepsis. Crit Care Med. (2019) 47(10):1433–41. 10.1097/CCM.0000000000003931 31385882 PMC7341116

[14] Weiss SL Zhang D Bush J Graham K Starr J Murray J Mitochondrial dysfunction is associated with an immune paralysis phenotype in pediatric sepsis. Shock. (2020) 54(3):285–93. 10.1097/SHK.0000000000001486 31764621 PMC7325426

[15] Goldstein B Giroir B Randolph A, Sepsis ICCoP. International pediatric sepsis consensus conference: definitions for sepsis and organ dysfunction in pediatrics. Pediatr Crit Care Med. (2005) 6(1):2–8. 10.1097/01.PCC.0000149131.72248.E6 15636651

[16] Wetzel RC. Pediatric intensive care databases for quality improvement. J Pediatr Intensive Care. (2016) 5(3):81–8. 10.1055/s-0035-1568146 31110890 PMC6512415

[17] Leteurtre S Duhamel A Grandbastien B Proulx F Cotting J Gottesman R Daily estimation of the severity of multiple organ dysfunction syndrome in critically ill children. CMAJ. (2010) 182(11):1181–7. 10.1503/cmaj.081715 20547715 PMC2917930

[18] Pollack MM Patel KM Ruttimann UE. PRISM III. Crit Care Med. (1996) 24(5):743–52. 10.1097/00003246-199605000-00004 8706448

[19] van Keulen JG Polderman KH Gemke RJ. Reliability of PRISM and PIM scores in paediatric intensive care. Arch Dis Child. (2005) 90(2):211–4. 10.1136/adc.2003.046722 15665184 PMC1720273

[20] Fiser DH. Assessing the outcome of pediatric intensive care. J Pediatr. (1992) 121(1):68–74. 10.1016/S0022-3476(05)82544-2 1625096

[21] Pollack MM Holubkov R Glass P Dean JM Meert KL Zimmerman J Functional status scale: new pediatric outcome measure. Pediatrics. (2009) 124(1):e18–28. 10.1542/peds.2008-1987 19564265 PMC3191069

[22] Meert KL Reeder R Maddux AB Banks R Berg RA Zuppa A Trajectories and risk factors for altered physical and psychosocial health-related quality of life after pediatric community-acquired septic shock. Pediatr Crit Care Med. (2020) 21(10):869–78. 10.1097/PCC.0000000000002374 32667767 PMC9059316

[23] John CC Opika-Opoka R Byarugaba J Idro R Boivin MJ. Low levels of RANTES are associated with mortality in children with cerebral malaria. J Infect Dis. (2006) 194(6):837–45. 10.1086/506623 16941352

[24] Ng PC Li K Leung TF Wong RP Li G Chui KM Early prediction of sepsis-induced disseminated intravascular coagulation with interleukin-10, interleukin-6, and RANTES in preterm infants. Clin Chem. (2006) 52(6):1181–9. 10.1373/clinchem.2005.062075 16613997

[25] Cavaillon JM Adib-Conquy M Fitting C Adrie C Payen D. Cytokine cascade in sepsis. Scand J Infect Dis. (2003) 35(9):535–44. 10.1080/00365540310015935 14620132

[26] Cavusoglu E Eng C Chopra V Clark LT Pinsky DJ Marmur JD. Low plasma RANTES levels are an independent predictor of cardiac mortality in patients referred for coronary angiography. Arterioscler Thromb Vasc Biol. (2007) 27(4):929–35. 10.1161/01.ATV.0000258789.21585.76 17255538

[27] Kachmar AG Watson RS Wypij D Perry MA Curley MAQ, Team REoSTfRFRI. Association of socioeconomic Status with postdischarge pediatric resource use and quality of life. Crit Care Med. (2022) 50(2):e117–e28. 10.1097/CCM.0000000000005261 34495879 PMC8810731

[28] Vassar M Holzmann M. The retrospective chart review: important methodological considerations. J Educ Eval Health Prof. (2013) 10:12. 10.3352/jeehp.2013.10.12 24324853 PMC3853868

[29] Watson RS Asaro LA Hertzog JH Sorce LR Kachmar AG Dervan LA Long-term outcomes after protocolized sedation versus usual care in ventilated pediatric patients. Am J Respir Crit Care Med. (2018) 197(11):1457–67. 10.1164/rccm.201708-1768OC 29313710 PMC6005554

[30] Sankar J Moodu S Kumar K Sankar MJ Kabra SK Lodha R. Functional outcomes at 1 year after PICU discharge in critically ill children with severe sepsis. Pediatr Crit Care Med. (2021) 22(1):40–9. 10.1097/PCC.0000000000002592 33031352

[31] Carlton EF Weeks HM Dahmer MK Quasney MW Sapru A Curley MAQ Inflammatory biomarkers are associated with a decline in functional status at discharge in children with acute respiratory failure: an exploratory analysis. Crit Care Explor. (2021) 3(7):e0467. 10.1097/CCE.0000000000000467 34278308 PMC8280074

[32] Fiser DH Long N Roberson PK Hefley G Zolten K Brodie-Fowler M. Relationship of pediatric overall performance category and pediatric cerebral performance category scores at pediatric intensive care unit discharge with outcome measures collected at hospital discharge and 1- and 6-month follow-up assessments. Crit Care Med. (2000) 28(7):2616–20. 10.1097/00003246-200007000-00072 10921604

