# Supplementary material for: Association of inflammatory biomarkers with new functional morbidity at hospital discharge in children who survive severe sepsis
Source: Front Pediatr. 2025 Mar 7;13:1519246. doi: 10.3389/fped.2025.1519246 (PMC11925794; doi:10.3389/fped.2025.1519246)
Supplement: Supplementary file 1 [file Image1.pdf]

Supplementary Material

SUPPLEMENTAL FIGURE LEGENDS

Supplemental Figure 1: MitoPSe Follow-Up Flow Diagram
A flow diagram detailing the cohort included within the parent MitoPSe study, and the current follow-up study presented here. Of the 166 critically ill children with sepsis in the parent study, 119 survived to hospital discharge and provided appropriate consent for follow-up and/or secondary research.

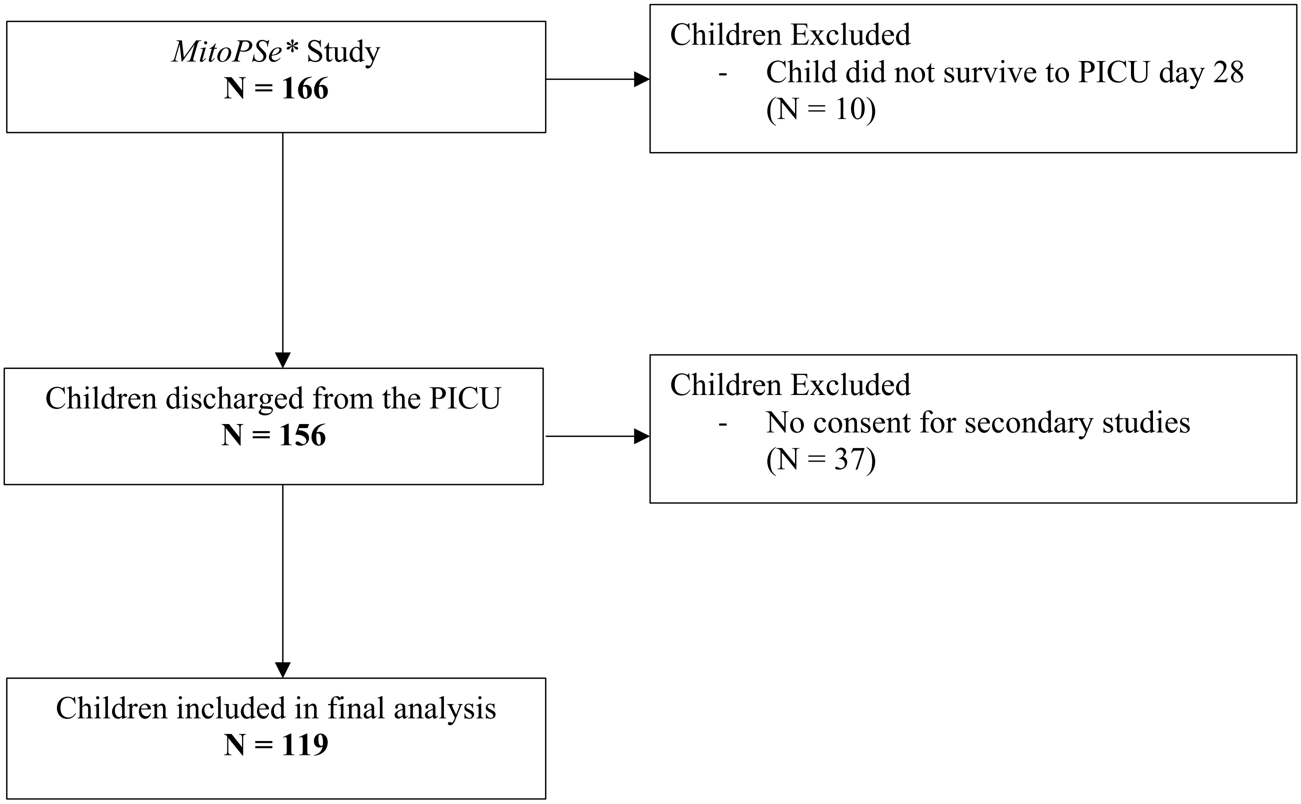

**MitoPSe* (Mitochondrial Dysfunction in Pediatric Sepsis) parent study
