# Supplementary material for: Association of inflammatory biomarkers with new functional morbidity at hospital discharge in children who survive severe sepsis
Source: Front Pediatr. 2025 Mar 7;13:1519246. doi: 10.3389/fped.2025.1519246 (PMC11925794; doi:10.3389/fped.2025.1519246)
Supplement: Supplementary file 2 [file Table1.docx]

**Supplemental Table 1. Assayed Inflammatory Biomarkers Included in Analysis**

| **Invitrogen**  Magnetic 30 Plex | IL-1B | MIP-1a | IFN-a |
| --- | --- | --- | --- |
|  | G-CSF | GM-CSF | IL-1RA |
|  | IL-10 | MIP-1B | TNF-a |
|  | IL-6 | MCP-1 | IL-2 |
|  | IL-12 | IL-15 | IP-10 |
|  | RANTES | IL-5 | IL-2R |
|  | sIL-2Ra | IFN-g | IL-4 |
|  | IL-8 |  |  |
| **Millipore**  Human Soluble Cytokine Receptor | sCD30 | sIL-6R | sRAGE |
|  | Sgp130 | sIL-1R | sIL-1RII |
| **R&D Systems**  Human Pre-Mixed Analyte (2 plex)  Human Magnetic Luminex Screening Assay (7 plex) | CD163 | IL-18 | CRP |

**Inflammatory biomarkers:** Interleukin-1 beta (IL-1B); Macrophage Inflammatory Protein-1 alpha (MIP-1a); Interferon-alpha (IFN-a); Granulocyte-Macrophage Colony-Stimulating Factor (GM-CSF); Interleukin-1 Receptor Antagonist (IL-1RA); Granulocyte Colony-Stimulating Factor (G-CSF); Macrophage Inflammatory Protein-1 beta (MIP-1B); Tumor Necrosis Factor-alpha (TNF-a); Interleukin-10 (IL-10); Monocyte Chemoattractant Protein-1 (MCP-1); Interleukin-2 (IL-2); Interleukin-15 (IL-15); Interleukin-6 (IL-6); Interferon Gamma-Induced Protein 10 (IP-10); Interleukin-12 (IL-12); Interleukin-5 (IL-5); Interleukin-2 Receptor (IL-2R); Regulated on Activation, Normal T Cell Expressed and Secreted (RANTES); Interleukin-4 (IL-4); Interleukin-8 (IL-8); Soluble CD30 (sCD30); Soluble Interleukin-6 Receptor (sIL-6R); Soluble Interleukin-2 Receptor alpha (sIL-2Ra); Soluble Receptor for Advanced Glycation End-products (sRAGE); Soluble Glycoprotein 130 (sgp130); Soluble Interleukin-1 Receptor I (sIL-1RI); Soluble Interleukin-1 Receptor II (sIL-1RII); Cluster of Differentiation 163 (CD163); Interleukin-18 (IL18); Interferon-gamma (IFNg); and C-Reactive Protein (CRP).
