# Supplementary material for: Association of inflammatory biomarkers with new functional morbidity at hospital discharge in children who survive severe sepsis
Source: Front Pediatr. 2025 Mar 7;13:1519246. doi: 10.3389/fped.2025.1519246 (PMC11925794; doi:10.3389/fped.2025.1519246)
Supplement: Supplementary file 3 [file Table2.docx]

**Supplemental Table 2 – Post-Sepsis Resource Utilization**

|  | New Functional Morbidity Present  (N=38) | New Functional Morbidity Absent  (N=81) |
| --- | --- | --- |
| **Readmission at 6-months Source, N (%)** |  |  |
| Infectious source | 8 (38.1) | 33 (70.2) |
| Emergent readmission | 13 (61.9) | 42 (89.4) |
| Planned readmission | 10 (47.6) | 1 (2.1) |
| **New Medications at Discharge Reason, N (%)** |  |  |
| Pain | 14 (42.4) | 10 (16.7) |
| Iatrogenic withdrawal | 3 (9.1) | 11 (18.3) |
| Gastrointestinal | 13 (39.4) | 12 (20.0) |
| Cardiovascular | 7 (21.2) | 4 (6.7) |
| Blood related issue | 7 (21.2) | 3 (5.0) |
| Mental Health | 1 (3.0) | 1 (1.7) |
| Sleep | 0 (0) | 1 (1.7) |
| Neurological (seizures) | 10 (30.3) | 7 (11.7) |
| Musculoskeletal | 0 (0) | 0 (0) |
| Oncological | 5 (15.2) | 0 (0) |
| Immunological | 3 (9.1) | 3 (5.0) |
| Urinary | 0 (0) | 1 (1.7) |
| Hormonal/endocrine | 5 (15.2) | 9 (15.0) |
| Gastrointestinal | 2 (6.1) | 6 (10.0) |
| Immunological | 12 (36.4) | 30 (50.0) |
| Respiratory | 5 (15.2) | 11 (18.3) |
| Corticosteroids | 2 (6.1) | 5 (8.3) |
| Sleep | 1 (3.0) | 0 (0) |
| Renal | 1 (3.0) | 0 (0) |
| **New Equipment at Discharge Type, N (%)** |  |  |
| Mechanical ventilation | 5 (16.7) | 7 (33.3) |
| Tracheostomy | 0 (0) | 2 (9.5) |
| Oxygen | 2 (6.7) | 2 (9.5) |
| Central venous catheter | 6 (20.0) | 4 (19.0) |
| Total Parental Nutrition | 4 (13.3) | 1 (4.8) |
| Feeding tube | 16 (53.3) | 5 (23.8) |
| Urinary device | 0 (0) | 2 (9.5) |
| Dialysis | 0 (0) | 0 (0) |
| Wheelchair | 5 (16.7) | 0 (0) |
| Prostheses/braces | 4 (13.3) | 1 (4.8) |
| Medication delivery | 2 (6.7) | 0 (0) |
| Drainage device | 0 (0) | 2 (9.5) |
| Respiratory | 1 (3.3) | 1 (4.8) |
| Endocrine | 2 (6.7) | 2 (9.5) |
| Gastrointestinal | 2 (6.7) | 0 (0) |
| Mobility aids | 1 (3.3) | 0 (0) |
| Monitoring device | 0 (0) | 3 (14.3) |
| **New Medical Services at Discharge Type, N(%** |  |  |
| Adolescent medicine | 0 (0) | 1 (1.8) |
| Allergist | 0 (0) | 3 (5.5) |
| Audiologist | 1 (2.9) | 1 (1.8) |
| Cardiologist | 5 (14.3) | 6 (10.9) |
| Dermatology | 2 (5.7) | 0 (0) |
| Developmental specialist | 0 (0) | 0 (0) |
| Endocrinologist | 6 (17.1) | 13 (23.6) |
| Gastrointestinal | 6 (17.1) | 9 (16.4) |
| Geneticist | 1 (2.9) | 0 (0) |
| Hematological | 4 (11.4) | 4 (7.3) |
| Immunological or Infectious Disease | 9 (23.7) | 7 (8.6) |
| Nephrology | 2 (5.7) | 2 (3.6) |
| Neurologist | 8 (22.9) | 9 (16.4) |
| Nutritionist | 0 (0) | 2 (3.6) |
| Oncology | 5 (14.3) | 1 (1.8) |
| Ophthalmology | 6 (17.1) | 2 (3.6) |
| Otorhinolaryngologist | 1 (2.9) | 3 (5.5) |
| Orthopedics | 4 (11.4) | 1 (1.8) |
| Physiatrist | 11 (31.4) | 2 (3.6) |
| Podiatrist | 0 (0) | 0 (0) |
| Psychiatrist | 3 (8.6) | 1 (1.8) |
| Psychologist | 1 (2.9) | 0 (0) |
| Pulmonologist | 5 (14.3) | 13 (23.6) |
| Rheumatologist | 0 (0) | 3 (5.5) |
| Social worker | 0 (0) | 0 (0) |
| Surgeon | 5 (14.3) | 4 (7.3) |
| Other | 7 (20.0) | 12 (21.8) |
| **New Outpatient Services at Discharge Type, N(%)** |  |  |
| Home health care nursing services | 7 (20.6) | 5 (23.8) |
| Homecare medical supplies | 6 (17.6) | 5 (23.8) |
| Physical therapy | 21 (61.8) | 10 (47.6) |
| Occupational therapy | 18 (52.9) | 6 (28.6) |
| Speech therapy | 11 (32.4) | 6 (28.6) |
| Alternative therapies | 0 (0) | 0 (0) |
| Day Hospital | 2 (5.9) | 0 (0) |
| Early Intervention | 2 (5.9) | 1 (4.8) |
| Inpatient rehab | 10 (29.4) | 2 (9.5) |
| Other | 2 (5.9) | 2 (9.5) |
